# Fall Detection of Elderly People Using the Manifold of Positive Semidefinite Matrices

**DOI:** 10.3390/jimaging7070109

**Published:** 2021-07-06

**Authors:** Abdessamad Youssfi Alaoui, Youness Tabii, Rachid Oulad Haj Thami, Mohamed Daoudi, Stefano Berretti, Pietro Pala

**Affiliations:** 1 ADMIR Laboratory, Rabat IT Center, IRDATeam, ENSIAS, Mohammed V University in Rabat, Rabat 10000, Morocco; youness.tabii@ensias.um5.ac.ma (Y.T.); or rachid.ouladhajthami@ensias.um5.ac.ma (R.O.H.T.); 2 MT Lille Douai, Institut Mines-Télécom, Centre for Digital Systems, F-59000 Lille, France; mohamed.daoudi@imt-lille-douai.fr; 3 CNRS, Centrale Lille, Institut Mines-Télécom, UMR 9189 CRIStAL, University Lille, F-59000 Lille, France; 4 Department of Information Engineering, University of Florence, 50121 Florence, Italy; stefano.berretti@unifi.it (S.B.); pietro.pala@unifi.it (P.P.)

**Keywords:** fall detection, healthcare, positive semidefinite matrices, Riemann manifold, Dynamic Time Warping, Gram matrix

## Abstract

Falls are one of the most critical health care risks for elderly people, being, in some adverse circumstances, an indirect cause of death. Furthermore, demographic forecasts for the future show a growing elderly population worldwide. In this context, models for automatic fall detection and prediction are of paramount relevance, especially AI applications that use ambient, sensors or computer vision. In this paper, we present an approach for fall detection using computer vision techniques. Video sequences of a person in a closed environment are used as inputs to our algorithm. In our approach, we first apply the V2V-PoseNet model to detect 2D body skeleton in every frame. Specifically, our approach involves four steps: (1) the body skeleton is detected by V2V-PoseNet in each frame; (2) joints of skeleton are first mapped into the Riemannian manifold of positive semidefinite matrices of fixed-rank 2 to build time-parameterized trajectories; (3) a temporal warping is performed on the trajectories, providing a (dis-)similarity measure between them; (4) finally, a pairwise proximity function SVM is used to classify them into fall or non-fall, incorporating the (dis-)similarity measure into the kernel function. We evaluated our approach on two publicly available datasets URFD and Charfi. The results of the proposed approach are competitive with respect to state-of-the-art methods, while only involving 2D body skeletons.

## 1. Introduction

In 2019, the United Nations (UN) published statistics about the world population [1]. According to this report, in the next years, the percentage of elderly people will grow considerably in Sub-Saharan Africa, Northern Africa, Western Asia, Latina America, Caribbean, Australia, North America, etc.In the same document, the estimated change in the percentage of elderly people between 2019 and 2050 is also reported. For example, the number of persons over 65 years in Morocco is expected to increase from 7.3% of population in 2019 to 11.2% of population in 2030.

On the other hand, the World Health Organization (WHO) published a report about the problems caused by falls [2]. This article reports an impressive statistic, according to which it is expected that most unintentional injuries in elderly people will be caused by falls. Another statistic shows that more than (646,000) persons die every year as a consequence of falling, and elderly people contribute the highest percentage of these deaths. They expect more than 37.3 million falls, the majority of them causing serious problems to healthcare services. Furthermore, the Center for Disease Control Prevention [3] showed statistics about adult and senior falls. It results that about 20% of falls caused serious consequences, e.g., fractures, head injuries or hip fractures (in more than 95% of falls). Overall, more than 3 million seniors enter emergency departments every year due to falls.

In the last few years, a lot of solutions have been developed to decrease the danger caused by falls. For example, there are a lot of works aiming to monitor persons by using cameras, sensors and ambient/fusion [4,5,6,7]. These methods analyze the motion of persons and aim to distinguish between falls and daily activities.

Several approaches use wearable sensors, such as accelerometers and gyroscopes to detect posture and inactivity of the person [8,9,10,11,12,13,14,15] and extract different features from the data: angles, directions, acceleration, etc. The classification step is typically performed by using thresholds or machine learning algorithms. Other works based their models on utilizing room information, such as sound and floor vibration [16,17]. Generally, it is very easy to setup a fall detection system using a wearable device-based approach. However, these systems are expensive, not robust and consume batteries. Moreover, the accuracy and intrusion of these methods depend on the specific scenarios. Last, it is impossible for the care services to visualize and verify the data to better understand and improve the obtained accuracy.

Computer-vision approaches monitor an imaged subject by using cameras [18,19,20,21,22,23,24,25,26,27,28,29,30,31]. They analyze the change of body shape by computing different features, such as the ratio between height and width of the box surrounding the person, the histogram projection of the silhouette, the coordinates of an ellipse surrounding the person and the key joints of the person’s skeleton. Furthermore, computer-vision approaches are the most used to detect fall thanks to their robustness and ease of setting up a fall detection system. These methods are highly accurate and do not depend on scenarios. In Section 2, we present more details about previous fall detection works appeared in the literature.

In this paper, we present an algorithm to detect fall using a computer-vision approach that does not rely on wearable sensors or handheld devices. Firstly, we apply the V2V-PoseNet [32] model to detect the skeleton of the person in a 2D image. In the second step, we measure the similarity between sequences of skeletons and use the matrix of similarity scores between all sequences as an input to a classifier. In doing so, we rely on the method in [33] and employ the Riemannian manifold of positive semidefinite matrices to compute a trajectory from the skeletons of the person. Then, we employ the Dynamic Time Warping algorithm to align the trajectories and compute the similarity scores between sequences. Finally, we use a Support Vector Machine (SVM) to classify between fall and non-fall events using the similarity scores.

The rest of the paper is organized as follow. In Section 2, we summarize works in the literature that detect fall by using ambient/fusion, wearable sensors and computer vision. In Section 3, we show the different operations that constitute our approach. In Section 4, we present the results of applying our solution by using the Chari and the URFD datasets and compare with state-of-the-art methods. Finally, in Section 5, we conclude our paper and present some perspectives for future work.

## 2. Related Work

In the last few years, many approaches have been proposed to detect fall of elderly people [4,5,6,7]. These works can be grouped in three main categories [4]: (*i*) methods that use wearable sensors to monitor the person and detect abnormal activities during time; (*ii*) solutions that use ambient/fusion to collect room information such as floor vibration and sound, with recent works that also utilize other technologies such as smartphones, Wi-Fi, etc.; and (*iii*) methods that use a camera to detect the change of body shape during time. In the following, we focus more on the methods in the third category since they are closer to our proposed approach.

**Wearable sensors**: Wearable device-based approaches use triaxial gyroscopes [12,13], accelerometers [8,9,10,11,15,34,35] or both types of sensors [36] to monitor the person and detect posture changes and inactivity. In these solutions, data acquired by the sensors are used to compute different features, such as angles [9,12], differences and derivatives of the sum *X*, *Y* and directions [8,9], maximum acceleration and fluctuation frequency [12], decreasing of heat rates [10], variation of different parts of the body [11], the acceleration of the body parts [13], mutual information and removing highly correlated features using Pearson correlation coefficient and Boruta algorithm [35], etc.In addition, they distinguish fall and non-fall events by using thresholds [8,9,10], machine learning [11,12,13,14,35] and deep learning algorithms [15,34].

**Ambient/fusion**: Many works used the sound captured in the environment as a clue for detecting the fall of a person [16,17]. This is obtained by detecting the sound of the person during fall and normal activities, in order to compute the Mel-frequency spectral coefficient. In the last step, fall and non-fall events are classified by using machine learning techniques.

**Computer vision-based**: Many methods have been developed to monitor a person using cameras. Sequences of frames are used to calculate different features such as histogram projection of the person’s silhouette [18,22,37]; aspect ratio and orientation of principal components [21]; motion vectors of the person [19,20], bounding box coordinates [22,24], feet-related features such as step length symmetry, normalized step count, speed and foot flat ratios [37]; body-related features such as amount of movement in the left and right side of the body, movement symmetry, shift in the centre of gravity and torso orientation [37]; etc.Other works employed Riemannian manifolds to analyze the shape of the person and detect fall [23,24]. In addition, solutions based on deep learning algorithms such as Convolution Neural Networks (CNNs) [19,20] and Long-Short Term Memory networks (LSTM) [38] have been also used.

Several methods exist that use the skeleton of the monitored person to compute features in every frame of a sequence. These methods can either detect the skeleton of a person in 2D images by using CNN models, such as OpenPose, PoseNet, ALphaNet, etc., or they can detect the skeleton in images captured by a Kinect sensor. Relying of the detected skeleton, several methods estimate the human pose by extracting features from the skeleton and classifying them. For example, Chen et al. [25] developed an algorithm to recognize accidental falls by using the skeleton information. They first detected the skeleton of the person by applying the OpenPose algorithm. Then, they computed the speed of descent of the hip joint’s center, the angle between the floor and the center-line of the human body and the ratio between the width and height of the rectangle surrounding the human body. They take on consideration the standing up of the person after fall in their algorithm. Their model achieved a success rate in fall recognition of 97%. Alaoui et al. [39] developed an algorithm to detect falls by using the variation of a person’s skeleton into the video. Firstly, they detected the joints of the person into the video by using OpenPose. Then, they computed the angle and the distance between the same joint into two sequential frames. Finally, they trained their model using SVM, KNN, Decision Tree and Random Forest to classify fall and non-fall sequences. The SVM classifier resulted the most effective in their work. Loureiro and Correia [40] employed the VGG-19 architecture to classify pathological gaits or to extract features from the skeleton energy image. After that, they fed these features into a linear Discriminant Analysis Model and Support Vector Machine to classify normal, diplegic and hemiplegic gaits simulated by healthy people.

There are many methods that use images captured by Kinect sensors, in order to generate joint positions of the human’s body. For example, Yao et al. [26] developed an algorithm that includes three steps. Firstly, they captured motion information of joints in 3D coordinates using the R, G and B channels of a pixel. They focused on a reduced set of 25 joints of the human skeleton. Then, every frame is encoded independently as a slice of motion image, in order to overcome the problem of losing information caused by trajectory overlap. In the last step, the Limit Of Stability Test (LOST) is used to detect fall from the start to the end key frame. They reported an accuracy of 97.35% on the TST v2 dataset, with effective performed reported also on the UT-A3D dataset. Kawatsu et al. [27] proposed an approach to detect fall. They developed two algorithms that use the skeleton generated from the Kinect SDK. The first algorithm aims to determinate the maximum distance between the floor and the position of all the joints. They detected fall by comparing this distance with a threshold. The second algorithm computes the average velocity of all the joints. In addition, in this case, to detect falls, the average velocity is compared with a threshold.

Alazrai et al. [28] developed a fall detection algorithm based on a representation layer and two classification layers. They used a Kinect sensor to collect RGBD images and derive 3D joint positions. They computed the Motion Pose Geometric Descriptor (MPGD) for every input frame in order to describe motion and pose of human body parts. After that, they employed an SVM to classify every frame in the first classification layer. The second classification layer employed the Dynamic Time Warping algorithm to classify fall and non-fall sequences generated from the SVM. They tested the model by using the 66-dataset that contains 14,400 frames and 180 activity sequences. Using five-fold cross validation, they achieved 98.01% precision, 97.13% recall and 97.57% F1-measure. Pathak et al. [29] proposed a fall detection method. They first detected and tracked key joints from a Kinect sensor and extracted two parameters using key joints. Then, to detect falls, they compared these parameters with a threshold. They also integrated in their system an alert message, which is sent to a predefined number when a fall event is detected. They tested the model on a real dataset of 50 persons, obtaining 94.65% accuracy in indoor environment. Abobakret et al. [31] presented an algorithm to detect fall using a skeleton posture and activity recognition. They analyzed local variations in depth pixels to recognize the posture using frame acquired from a Kinect-like sensor. They employed a random decision forest to distinguish standing, sitting and fall postures and detected fall events by employing an SVM classifier. They reported 99% sensitivity on a synthetic live dataset, 99% sensitivity on a synthetic dataset and 96% sensitivity on popular live dataset without using accelerometer support. Seredin et al. [30] developed an algorithm to detect falls by using skeleton feature encoding and SVM. They computed a cumulative sum to combine the decision on a sequence of frames. The model achieved 95.8% accuracy in the cross validation procedure, using a Leave-One-Person-Out protocol.

**Discussion**: As summarized above, different methods exist for fall detection. These algorithms have been evaluated using their sensitivity and specificity, which resulted highly effective in many cases. For example, algorithms that employ depth sensors are very accurate. In addition, systems reported a high accuracy when they employed multi-dimensional combination between physiological and kinematic features [26,27,28,29,30,31]. However, existing solutions show several limitations. For example, systems that use Wearable [8,9,10,11,12,13,14,15,34,35,36] and ambient [16,17] features have some disadvantages, which are related to the inconceivability of visually checking object information. In addition, systems that use computer vision techniques [18,19,20,21,22,23,24,25] are flexible. The majority of these algorithms are not specific, do not depend on different scenarios, are simple to setup and are very accurate [4].

## 3. Proposed Method

**Figure 1:**
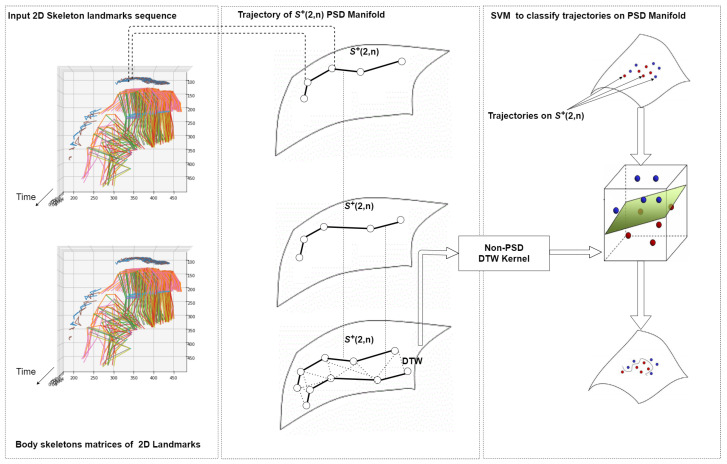
Overview of the proposed approach. After detecting the skeleton of a person in every frame of a sequence, a Gram matrix is computed for each frame. In this way, a sequence is represented by a trajectory of points on the manifold of positive semidefinite matrices $S^{+}\left(2,n\right)$. The Dynamic Time Warping (DTW) algorithm is employed to align trajectories on the manifold. Finally, a kernel generated from DTW and linear SVM are employed to classify fall and non-fall sequences.

We present a method to classify between fall and non-fall events, which is based on computing the similarity between video sequences. Figure 1 provides an overview of the proposed approach. As a preliminary step, we employ the V2V-PoseNet [32] model to extract a skeleton from each frame of a sequence. After that, we represent our data (i.e., the sequence of skeletons) using Gram matrices and thus on the Riemannian manifold of positive semidefinite matrices. To this end, we compute a Gram matrix from the skeleton in each frame of a sequence. Gram matrices are symmetric matrices that lay on the Riemannian manifold of positive semidefinite matrices, so that a sequence is transformed to a trajectory of points on the manifold, i.e., a point is derived on the manifold for each frame. A Riemannian metric is then defined on the manifold to compare two Gram matrices. Finally, the Dynamic Time Warping (DTW) algorithm is used to extend the Riemannian metric from the frame level to the sequence level and compute a similarity score between two sequences. This aims to be invariant with respect to differences in the speed of execution of the action captured in a sequence. This score is the input to a linear SVM classifier that we use to distinguish between fall and non-fall events.

### 3.1. Skeleton of a Person

**Figure 2:**
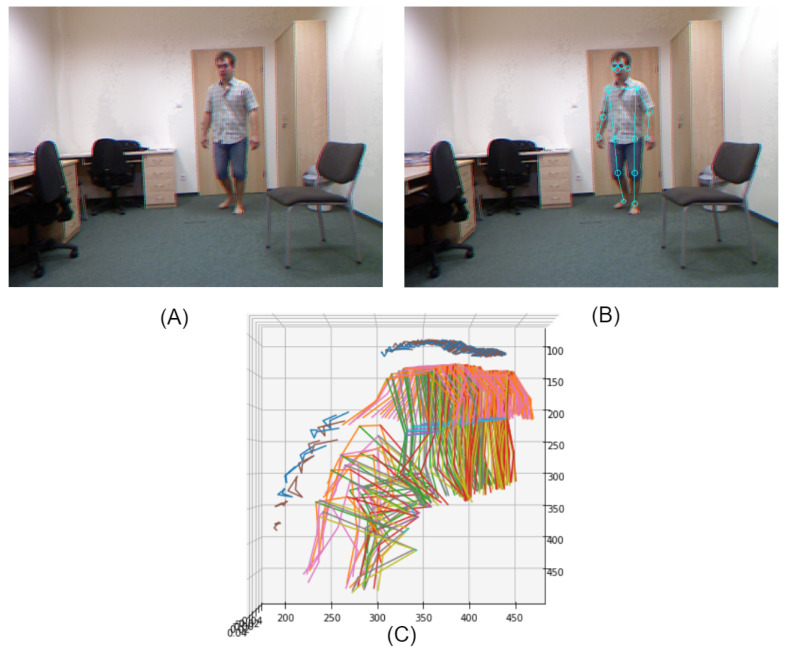
Results of applying the V2V-PoseNet model to detect the skeleton of a person: (**A**) the input frame to V2V-PoseNet; (**B**) projection into the input image of the skeleton detected by V2V-PoseNet; and (**C**) the 3D projection of a sequence of skeletons corresponding to a video sequence.

The skeleton of a person is detected by using the V2V-PoseNet [32] model for each frame of a sequence. There are four steps in the construction of the V2V-PoseNet model. First, a volumetric convolution is computed by utilizing a volumetric basic block, then a volumetric batch normalization [41] plus an activation operation are applied to remove negative values. In the second step, a volumetric residual block is employed to extend from a 2D convolution block. The residual blocks exploit the result of the previous convolution blocks in order to extract more features. The third step applies a down-sampling operation using max-pooling, thus reducing the image dimension and the processing time. In the last step, a volumetric decoding block is used to decode the results of the previous steps (i.e., decode features found in the previous steps and visualize them into the input images). This step also contains a volumetric normalization block and an activation operation (ReLu) to remove the negative values as well as to normalize the values produced after the decoder block. In summary, the V2V-PoseNet [32] model applies convolutional blocks and computes features, producing the pose confidence and joint key points of the person in a given image as initial result. Then, it visualizes these features into the input image as key points. We also tested other algorithms to detect the skeleton of the person such as OpenPose and AlphaNet. We found that V2V-PoseNet is the most accurate to detect skeletons from videos. Figure 2 illustrates the results of detecting skeletons by using V2V-PoseNet and the 3D projection of a sequence of skeletons detected from a video sequence.

In our work, we aim at detecting falls by analyzing the change of a person’s body during a sequence. To this end, we extract the skeleton of the person and represent the shape by a set of points. In this way, the shape is given by a time series of the 2D coordinates of the points. This time series contains the coordinates of all the skeletons tracked during an event. A fall event is detected from a sequence containing *m* frames, where every frame is represented by a vector with the skeleton coordinates, i.e., the vector $V_{i}$ contains $\left\{\left(x_{1},y_{1}\right),\cdots ,\left(x_{n},y_{n}\right)\right\}$, where *n* is the number of joints. Every video is characterized by a set of vectors, where every vector represents the coordinates of skeleton’s points (i.e., a video corresponds to $V_{1},\cdots ,V_{m}$). More specifically, every $V_{1\leq i\leq m}$ is a n×2 matrix.

We represent a sequence of skeletons by a sequence of Gram matrices, where each Gram matrix is computed as:

(1) $$G_{i}=V_{i}V_{i}^{T}\;.$$

The resulting matrix is an n×n positive semidefinite matrix, with a rank smaller than or equal to 2. Such n×n positive semidefinite matrices of rank 2 have been studied in several works [42,43,44,45,46,47,48].

These Gram matrices belong to $S^{+}\left(2,n\right)$, the manifold of n×n positive semidefinite matrices of rank 2 for which a valid Riemannian metric can be defined as follows:

(2) $$d\left(G_{1},G_{2}\right)={\left[\mathrm{tr}\left(G_{1}\right)+\mathrm{tr}\left(G_{2}\right)-2\mathrm{tr}\left({\left(G_{1}^{\frac{1}{2}}G_{2}G_{1}^{\frac{1}{2}}\right)}^{\frac{1}{2}}\right)\right]}^{\frac{1}{2}},$$

being $G_{1}$ and $G_{2}$ two generic Gram matrices in $S^{+}\left(2,n\right)$. We can also use Singular Value Decomposition (SVD) to compute the previous distance by employing:

(3) $$d\left(G_{1},G_{2}\right)=min_{Q\in Q^{d}}\left|\right|V_{1}Q-V_{2}{\left|\right|}_{F}\;.$$

The optimal value $Q^{*}=AU$ is computed by using SVD, where $V_{1}^{T}V_{2}=A\Sigma U^{T}$.

Our final goal is classifying fall and non-fall sequences by using the similarity scores. This requires for a method computing a similarity score between sequences, as described in the following.

### 3.2. Sequence Similarity Using the DTW Algorithm

Dynamic Time Warping (DTW) is an algorithm that aims at measuring the similarity between two temporal sequences. As such, it is largely employed in time series analysis. One important characteristic of DTW is its capability of computing the similarity between two sequences that vary in speed, so with different acceleration or deceleration. For example, two sequences can correspond to two persons walking with different velocities.

In fall detection, the major difference between a fall event and a non-fall event is the acceleration and deceleration of a person. The acceleration of a falling person is bigger than the acceleration of a person who is not falling. For this reason, we employ DTW to measure the similarity between sequences [49,50].

As discussed above, we represent a sequence of skeletons as a sequence of Gram matrices. For example, let $V^{1}=\left\{V_{0}^{1},\cdots ,V_{\tau_{1}}^{1}\right\}$ and $V^{2}=\left\{V_{0}^{2},\cdots ,V_{\tau_{2}}^{2}\right\}$ be two sequences of skeleton matrices. Computing the Gram matrices, we represent $V^{1}=\left\{V_{0}^{1},\cdots ,V_{\tau_{1}}^{1}\right\}$ by $G^{1}=\left\{G_{0}^{1},\cdots ,G_{\tau_{1}}^{1}\right\}$, where $G_{i}^{1}=V_{i}^{1}V_{i}^{1^{T}},0\leq i\leq \tau_{1}$, and $V^{2}=\left\{V_{0}^{2},\cdots ,V_{\tau_{2}}^{2}\right\}$ by $G^{2}=\left\{G_{0}^{2},\cdots ,G_{\tau_{2}}^{2}\right\}$, where $G_{j}^{2}=V_{j}^{2}V_{j}^{2^{T}}$ and $0\leq j\leq \tau_{2}$. Then, we compute the distance between any two pairs of Gram matrices in the two sequences. The result is a matrix of size $\tau_{1}\times \tau_{2}$, where D(i,j) is the distance between $G_{i}^{1}$ and $G_{j}^{2}$ (i.e., $V_{i}^{1}$ and $V_{j}^{2}$, respectively), $0\leq i\leq \tau_{1}$ and $0\leq j\leq \tau_{2}$. D(i,j) is computed as:

(4) $$D\left(i,j\right)=d\left(G_{i}^{1},G_{j}^{2}\right)\;.$$

where d(·,·) is the Riemannian metric defined in (2). The matrix *D* is used as input to the DTW algorithm that computes the distance $D_{DTW}\left(G^{1},G^{2}\right)$ between the two sequences of Gram matrices.

The result of this computation is used to evaluate the Gaussian kernel required to train the SVM classifier. For two generic sequences *i* and *j*, this is defined as:

(5) $$k\left(i,j\right)=\frac{1}{2}\times \exp-\frac{D_{DTW}\left(i,j\right)}{2\sigma^{2}}\;.$$

The DTW algorithm that we use here is based on the work of Gudmundsson et al. [51]. The Algorithm 1 summarizes the computation of the Gaussian Kernel using the Riemannian distance between two sequences.
**Algorithm 1:** Gaussian Kernel.
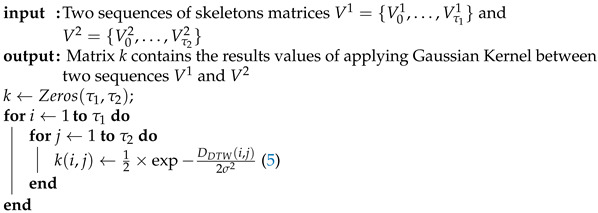


In the DTW algorithm, a matrix *M* is computed, where the M(i,j) element is the sum of k(i,j) and the minimum of M(i,j−1)+M(i−1,j−1)+M(i−1,j). The matrix *M* has size $\left(1+\tau_{1}\right)\times \left(1+\tau_{2}\right)$, where $\tau_{1}$ is the size of the sequence $V^{1}$, $\tau_{2}$ is the size of the sequence $V^{2}$, $1\leq i\leq 1+\tau_{1}$ and $1\leq j\leq 1+\tau_{2}$. In particular, the element M(i,j) is computed as:

(6) M(i,j)=k(i,j)+min{M(i,j−1),M(i−1,j−1),M(i−1,j)}.

The similarity score between these two sequences is the last value of the matrix (i.e., $M(1+\tau_{1},1+\tau_{2})$). The Algorithm 2 summarizes the DTW procedure.
**Algorithm 2:** Dynamic Time Warping.
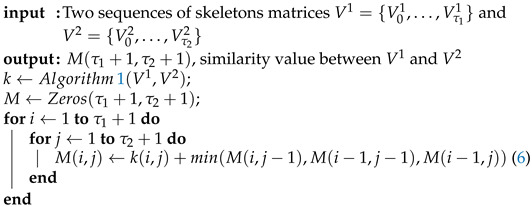


### 3.3. Classification

Once the similarity scores have been computed, we use them as input to an SVM classifier. To this end, we represent every sequence by a vector, which contains the similarity scores between this sequence and all the other sequences. Let $V^{i}=\left\{v_{0}^{i},\cdots ,v_{\tau_{i}}^{i}\right\}$ be a sequence of skeleton matrices, where $\tau_{i}$ is the number of skeleton matrices (respectively, $G^{i}=\left\{G_{0}^{i},\cdots ,G_{\tau_{i}}^{i}\right\}$). The similarity vector is computed as $\left\{\phi \left(V^{1},V^{i}\right),\cdots ,\phi \left(V^{i},V^{i}\right),\cdots ,\phi \left(V^{i},V^{s}\right)\right\}$, where *s* is the number of sequences, and $\phi {\left(V^{i},V^{j}\right)}_{0\leq i,j\leq s}$ is the similarity score (computed using the Gaussian kernel) between sequences $V^{i}$ and $V^{j}$. Now, we can represent the set of sequences by a set of vectors containing the similarity scores. This results into a matrix *X*, where $X_{j}$ is the *j*thline of matrix *X* and corresponds to the similarity scores between the *j*th and all others sequences (it also contains the similarity score between the *j*th sequence and itself.

The Algorithm 3 summarizes the computation of the similarity scores matrix.
**Algorithm 3:** Computation of the similarity scores matrix.
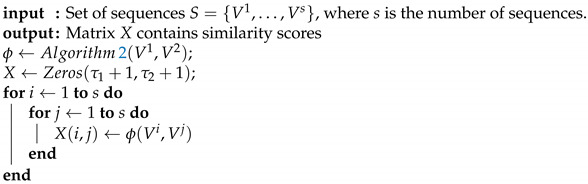


## 4. Experimental Results

We evaluated the performance of our approach on the Charfi [52] and UR Fall Detection [53] datasets, and compared the results against state-of-the-art methods as reported below.

### 4.1. Charfi Dataset

**Figure 3:**
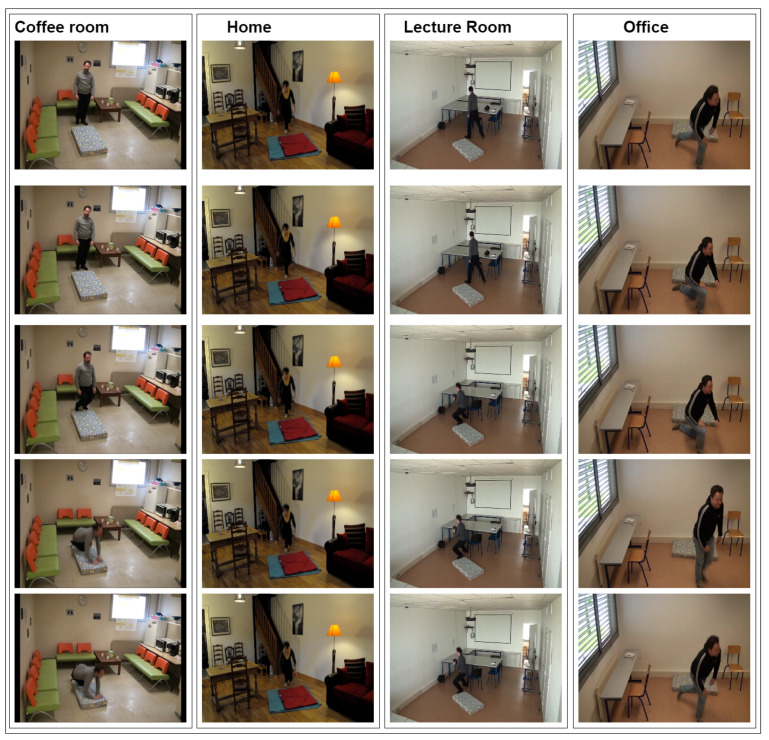
Charfi dataset: Examples frames taken from lecture room, home, coffee room and office locations.

This dataset was designed and acquired at the ”Laboratoire Electronique, Informatique and Image” (Le2i) [52]. It includes 240 videos with resolution of 320×240, each reporting fall and non-fall events as occurring in daily activities. The dataset includes several daily life activities, such as sitting, laying, sleeping and also falling events, while performed in different locations, including reading room, office, coffee and home. Various views of the camera are used to monitor the imaged person. The background of the video is fixed and simple, while the texture of images is difficult. Moreover, shadows are also present in this dataset. Every frame is labeled with the location, the fall/non-fall class and by the coordinates of the person given as bounding box. Figure 3 illustrates some frames from the Charfi dataset in different locations.

**Figure 4:**
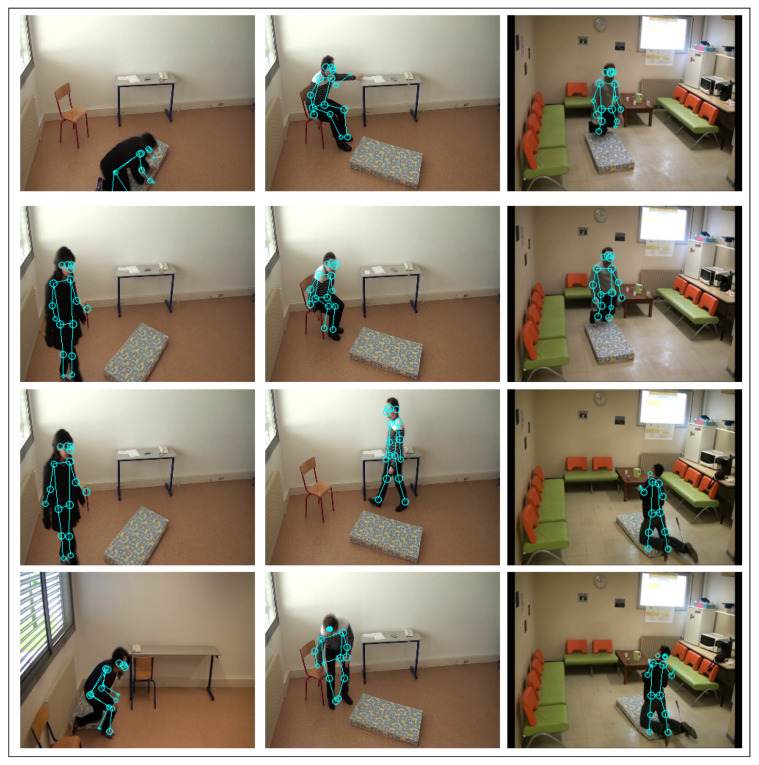
Charfi dataset: Skeleton detected by applying V2V-PoseNet to some frames.

Using the V2V-PoseNet, we first extract the skeleton joints of the person in the frames, as illustrated in Figure 4. Interestingly, the detection is robust to the presence of shadows in the image.

To evaluate the performance of our algorithm, we adopted a Leave-One-Out cross validation protocol as in [22,54]. According to this, the training set comprises all sequences except one, while the sequence left out is used as test. Using iteratively all the sequences of the Charfi dataset as test, we obtained the normalized confusion matrix reported in Table 1.

**Figure 5:**
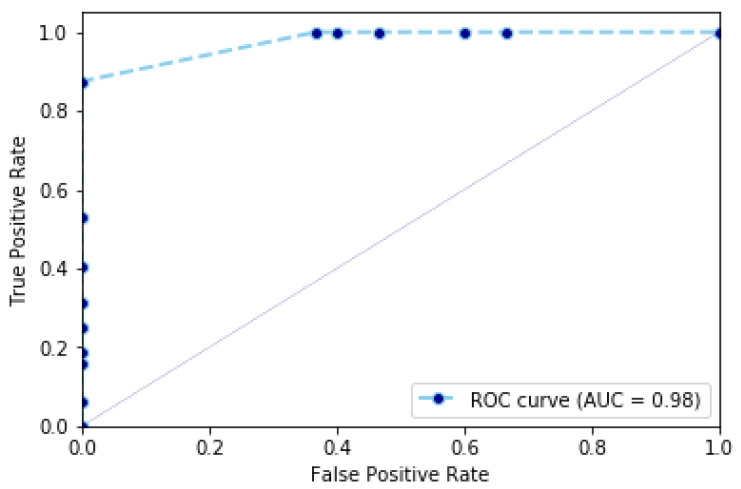
Charfi dataset: ROC curve representing the cumulative rate between true positive rate and false positive rate.

Table 2 illustrates the accuracy, specificity and sensitivity of our algorithm and other works on the Charfi dataset. For example, applying our algorithm results into an accuracy of 93.67%, a specificity of 87% and a sensitivity of 100%. In particular, we observe in this dataset there are some videos corresponding to a normal activity of the person that are similar to a fall sequence. For these sequences, the speed of the person is similar to the speed of a person as occurring in fall sequences. This makes the detection challenging and results in a specificity (i.e., the capability of classifying a non-fall sequence that is similar to a fall) of 87%. In addition, our algorithm classifies between fall and non-fall sequences with an accuracy of 93.67%. This value represents the capacity of classifying between fall sequences and non-fall sequences classified as non-fall sequences. This value is represented also by the ROC curve in Figure 5, which represents the cumulative rate between true positives (i.e., fall sequences classified as fall sequences) and false positives (i.e., non-fall sequences classified as such).

### 4.2. UR Fall Detection Dataset

**Figure 6:**
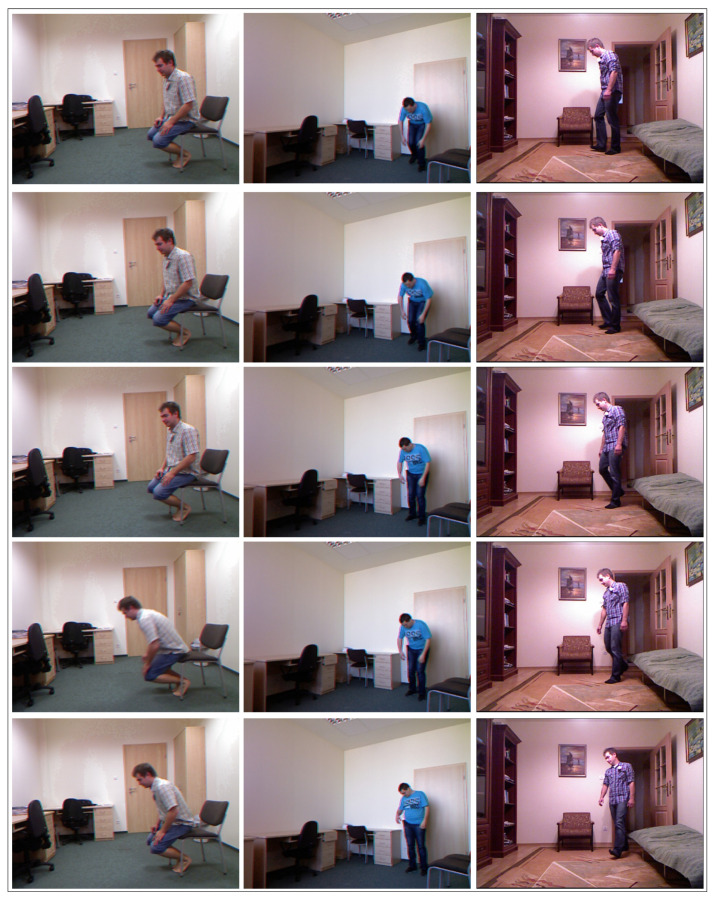
URFD dataset: example frames.

The URFD dataset whas designed and captured by the Interdisciplinary Center for Computational Modeling at the University of Rezeszow [53]. The imaged person is monitored in a closed environment, in order to capture the maximum of person’s activities. Daily activities were considered such as lying on the floor, crouching down, lying on the bed/couch, sitting down and picking up an object. Video sequences corresponding to fall events contain the person during fall, after fall (this part of the sequence is not used in the classification) and before fall. Figure 6 shows some example frames from the URFD dataset.

**Figure 7:**
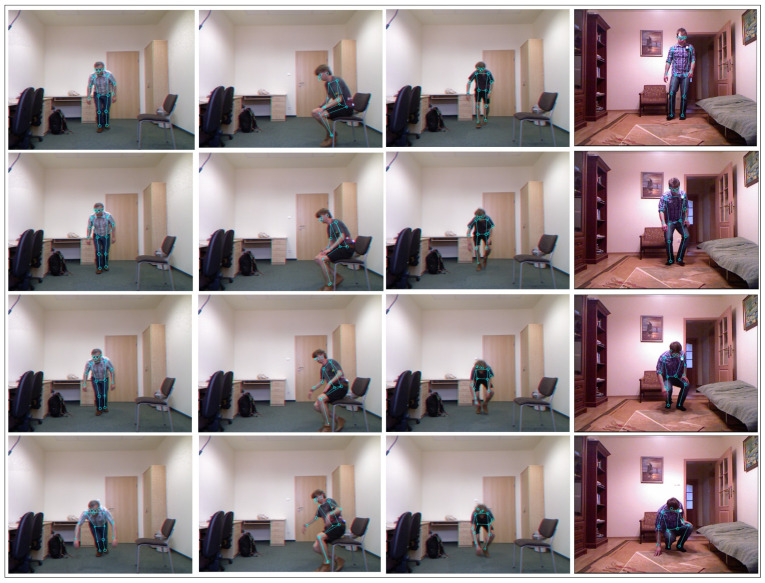
UR dataset: Skeleton detected with the V2V-PoseNet on some frames.

The results of extracting the skeleton joints of a person with the V2V-PoseNet model on some frame of the UR dataset are shown in Figure 7.

We evaluated the performance of using URFD by adopting a Leave-One-Out cross validation protocol. The concept of this validation protocol consists of using one sequence left out for testing, while all the remaining sequences are used for training. The normalized confusion matrix obtained by applying our approach on the URFD dataset is reported in Table 3.

**Figure 8:**
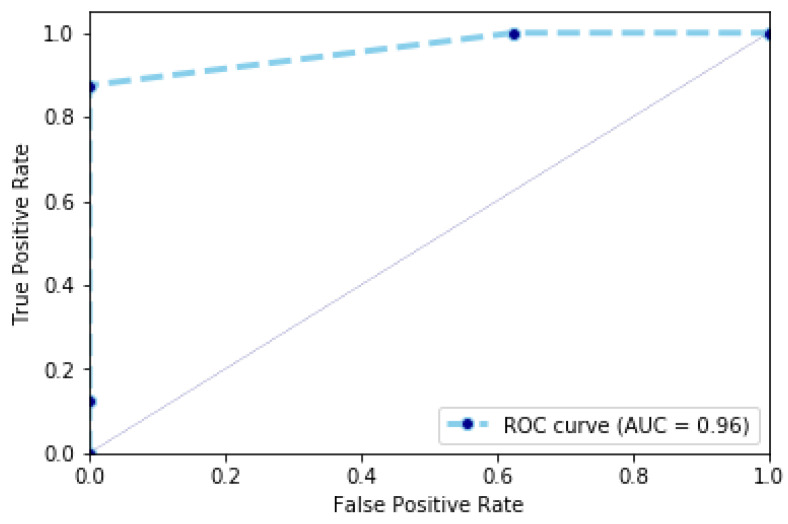
URFD dataset: ROC curve representing the cumulative rate between true positive rate and false positive rate.

Videos in the URFD dataset have a short duration and correspond to fall and non-fall sequences. For these videos, the speed of a person performing daily activities is not high, when compared to some videos in the Charfi dataset. This results in a higher specificity that reaches the value of 96.55%. This value is represented also using the Receiver Operating Characteristic (ROC) curve in Figure 8. The ROC curve is used to plot the probability of detecting fall by using similarity scores (the ratio between sequences, which are detected as fall sequences and all fall sequences) against probability of detecting non-fall sequences (the ratio between non-fall sequences, which are detected as fall sequences and all non-fall sequences) at various thresholds. The comparison between the results obtained by our proposed method and approaches reported in the state-of-the-art on the URFD dataset is shown in Table 4. The speed of the person has an important impact on the classification. For this reason, our algorithm reaches 100% sensitivity for both datasets. The most critical challenge for our algorithm is the difference in acceleration (deceleration) values. For example, a person who sits down very quickly is probably classified as a fall sequence. Moreover, the skeleton of the person will not be detected in dark room. This can suggest a possible adaptation of our algorithm with very poor lighting, in order to overcome the problem of low illumination.

### 4.3. Cross Data Evaluation

We also performed a cross-dataset evaluation. We trained our algorithm using sequences from the Charfi dataset. After that, we tested our model using sequences from the URFD dataset and vice versa. Table 5 illustrates the results of using the cross data evaluation protocol. Our algorithm has an accuracy of 87.39%, sensitivity of 100% and specificity of 62.5% using the Charfi dataset to train our algorithm and the URFD dataset to test it. In addition, our algorithm reports an accuracy of 85.34%, specificity of 62.85% and specificity of 95.06% by utilizing the URFD dataset to training and the Charfi dataset for testing. Our algorithm is able to detect fall sequences. For this reason, we found a high value for sensitivity. However, our method cannot detect non-fall sequences correctly. For this reason, we found a low value for specificity because of the differences between the length of non-fall sequences into the Charfi and URFD datasets. Charfi contains sequences with different conditions such as light and dark environment, different places, etc.; thus, we found that the accuracy using Charfi to train our model is greater than that using URFD.

### 4.4. Computation Time

In order to measure the overall processing time of our algorithm, we computed the time of every step included in our processing pipeline: (*i*) the time for extracting the skeleton from an individual frame; (*ii*) the time for applying the DTW algorithm between two sequences; and (*iii*) the time for the classification step (i.e., time to classify fall and non-fall sequences by a linear SVM). These times were computed on a laptop equipped with a i5-7200U (7th gen) processor, 8 GB of RAM and 2 GB NVIDIA GeForce 940MX.

Table 6 presents the results of computing the processing time for each step. We used two images to evaluate the time of the V2V-PoseNet step. For the first image taken from the Charfi dataset, we measured a time of 0.277 s to extract the skeleton. For the same step, but performed on an image from the URFD dataset, the skeleton detection operation required 0.32 s. This difference can be explained by the different resolution of the images in the Charfi and URFD datasets. It is relevant to note here that V2V-PoseNet is capable of detecting the skeleton without problem due to shadow.

The time for the DTW step was computed using two sequences from the Charfi dataset, the first one with 12 frames and the second containing 32 frames. The resulting time was 0.063 s. For the URFD dataset, we computed the DTW step for two sequences, with 12 and 30 frames, respectively, with a processing time of 0.061 s. The last step consists of classifying the similarity score vectors using sequences from the URFD dataset. The time required by this step was 0.053 s. For the Charfi dataset, the time taken for the classifying fall and non-fall sequences was 0.65 s. It can be noticed that the processing time is low, yet the approach is theoretically solid and robust.

### 4.5. Discussion

The proposed method aims to detect falls using a computer-vision approach. The major advantage of using a camera to monitor a person is overcoming the problem of background noise in the environment that is observed when using wearable sensors [8,9,10,11,12,13,15,34,35,36]. In addition, a computer-vision approach is very flexible because it does not depend on the particular scenario, it is not specific, it does not consume much time and it is simple to set up [4].

Our algorithm is based on using a CNN model to detect the person’s skeleton into every frame, which is similar to other works (e.g., [25,26,39]). With respect to other works in the literature, we compute different features from the skeleton of an imaged person. In particular, in our approach, we represent the sequence of skeletons by a set of Gramian matrices, which result into a trajectory of points on the Riemannian manifold of positive semidefinite matrices. After that, we employ a Riemannian metric, a Gaussian Kernel and the DTW algorithm, in order to compute similarity scores between sequences. In the last step, we employ a linear SVM to classify between fall and non-fall events using similarity scores. Using the skeleton of the person has the clear advantage of overcoming the noise, which can occur by removing the background [23]. We also employ the Riemannian manifold in a different way with respect to that employed in other works (e.g., [23]). In addition, our algorithm does not depend on the person’s information, such as color. This differs from other works in the literature that cannot detect the silhouette of the person when the color of the person’s clothes is similar to the background [22,23,57]. In addition, our approach is able to detect the skeleton of the person in a video sequence that contains other moving objects, in contrast to methods that detect the silhouette of the person by removing the background (e.g., [21,52,54,55,56,60]).

Our approach only takes into consideration changes in the person’s skeleton during a video sequence, or a difference in person acceleration during a fall and non-fall real life event. In addition, our approach does not depend on the position of the camera. Furthermore, our algorithm shows a high classification rate on the URFD and Charfi datasets, as reported in Table 2 and Table 4. The same tables also show our approach is competitive with respect to other state-of-the-art methods.

***Limitations***: Some problems still occur in our algorithm as in most of the computer-vision systems. Our algorithm aims to detect falls for a single person living alone at home, and it cannot manage multiple persons. In addition, our method detect sequences of daily activities as fall sequences, when the person’s acceleration is high. For this reason, it reports a specificity of 87% on Charfi and 93% on URFD. Furthermore, it is very difficult to detect the skeleton in dark environments using V2V-PoseNet.

From a more general point of view, fall detection research still suffers from some inherent limitations. The most evident one is related to the nature of the available data. The majority of fall detection datasets are small due to the number of participants. They also contain only a few simulated falls. For this reason, the validity of the test performed on such data is diminished and the reproducibility in real world scenarios needs to be proved. However, this seems to still be a difficulty that is problematic to remove. A further limitation of fall detection from simulated data is the inability to handle imbalanced datasets. In the real world, there are many more non-fall events than fall ones. Due to this, the accuracy is biased toward correct detection of non-fall events rather than correct detection of falls. In addition, the majority of fall detection datasets do not take into consideration objects that an aged person can employ such as crutches. For works based on background removal, it is necessary to take into consideration crutches. In our case, the crutch does not cause an occlusion problem, because the skeleton of the person can be detected with or without crutches.

## 5. Conclusions

In this paper, we present an algorithm to detect fall events in video sequences by using the manifold of positive semidefinite matrices. Our method consists of four steps. In the first step, the skeleton of the imaged person is extracted from every frame of a sequence. The sequence of skeletons is then represented on the manifold of positive semidefinite matrices, during the second step. After that, in the third step, we compute the similarity scores between sequences using the DTW algorithm with a Riemannian metric. In the last step, an SVM classifier with a linear kernel is used to classify between fall and non-fall sequences. In the experiments, we demonstrated that our method achieves results that are competitive with state-of-the-art solutions on the same datasets. As future work, we aim to extend our approach to data captured by IR cameras. To make our model dynamic, we will recompute the similarity scores matrix for each new video sequence.

## Figures and Tables

**Table 1:** Charfi dataset: The confusion matrix obtained by applying our approach.

		Predicted label	
		Fall	Non-Fall
Real label	Fall	97	0
	Non-Fall	19	131

**Table 2:** Charfi dataset: Sensitivity, specificity and accuracy of our work in comparison to state-of-the-art methods.

Methods	Sensitivity	Specificity	Accuracy
Georgios Goudelis et al. [54]	-	-	100–96.6
Charfi et al. [52]	73	97.7	-
M. Chamle et al. [55]	83.47	73.07	79.31
Arisa Poonsri et al. [21]	93	64.29	86.21
Alaoui et al. [56]	94.55	90.84	90.9
Alaoui et al. [39]	95	100	97.5
**Ours**	100	87	93.67

**Table 3:** URFD dataset: The confusion matrix obtained by applying our algorithm.

		Predicted label	
		Fall	Non-Fall
Real label	Fall	63	0
	Non-Fall	2	27

**Table 4:** URFD dataset: Sensitivity, specificity and accuracy of our work in comparison to state-of-the-art methods.

Methods	Sensitivity	Specificity	Accuracy
Ali, Syed Farooq et al. [57]	99.03–99.13	99.03	-
Kepski et al. [58]	100	96.67	95.71
Bourke et al. [59]	100	90	-
Kepski et al. [53]	100	92.5	95
Alaoui et al. [39]	100	95	97.5
Yixiao Yun et al. [23]	96.77	89.74	-
**Ours**	100	93	96.55

**Table 5:** Cross data evaluation: Sensitivity, specificity and accuracy using the Charfi dataset to train our algorithm and the URFD dataset as testing and vice versa.

Training Dataset	Testing Dataset	Sensitivity	Specificity	Accuracy
Charfi	URFD	100	62.5	87.39
URFD	Charfi	95.06	62.85	85.34

**Table 6:** Computation time (in seconds) for each step of our algorithm. Computation times were computed separately for the Charfi and URFD datasets.

Dataset	V2V-PoseNet	DTW	Classification (Linear SVM)
URFD	0.32	0.061	0.053
Charfi	0.277	0.063	0.65

## Abbreviations

The following abbreviations are used in this manuscript:

DTW	Dynamic Warping Time
SVM	Support Vectors Machine
WHO	Wold Heath Organization
ROC	Receiver Operating Characteristic
CNN	Convolutional Neural Network
